# COVID-19: Second Wave or Multiple Peaks, Natural Herd Immunity or Vaccine – We Should be Prepared

**DOI:** 10.1017/dmp.2020.349

**Published:** 2020-09-10

**Authors:** Rima Moghnieh, Dania Abdallah, Abdul Rahman Bizri

**Affiliations:** Division of Infectious Diseases, Department of Internal Medicine, Makassed General Hospital, Beirut, Lebanon; Division of Infectious Diseases, Hôtel Dieu de France, Beirut, Lebanon; The Lebanese Society of Infectious Diseases and Clinical Microbiology task force for COVID-19 pandemic; Pharmacy Department, Makassed General Hospital, Beirut, Lebanon; Division of Infectious Diseases, Department of Internal Medicine, American University of Beirut, Beirut, Lebanon

**Keywords:** comorbidities, contingency planning, COVID-19, herd immunity, preparedness, vaccination, vitamins, zinc

## Abstract

Between December 31, 2019, and August 30, 2020 (date of this article), the novel coronavirus and its corresponding infection, coronavirus disease (COVID-19), increased to more than 25 million cases, and 843 158 deaths have been registered. Countries around the world have been affected, albeit in different levels and intensities.

Despite implementations of preventive public health measures, most countries are seriously preparing for 1 or more waves. The threat of this surge is likely to persist until herd immunity is acquired either by natural infection or through vaccination. However, given the time frame needed for herd immunity to occur and the low probability that a vaccine will be available on a global scale by the coming fall and winter seasons, contingency preparedness plans should be established and put in place for the coming days or months. These plans should help mitigate new peaks of the pandemic while relaxing the social isolation rules, patient, public health, and hospital levels.

In this article, we discuss recommendations that practicing physicians and public health agencies should provide to individuals, especially those at risk of infection, to take and implement pre-emptive measures in anticipation of the potential next peak of the pandemic.

Since the second decade of the 20th century, the global pandemic caused by the 2019 novel coronavirus disease (COVID-19) has shaken the world in an unprecedented way. Almost all countries around the world have been affected, albeit in different intensities. Some countries have experienced high peaks, whereas others are still anticipating further increases in numbers of positive COVID-19 cases.^1^ Although the latter countries are still extremely concerned about an additional increase in their cases, countries that have passed their peak, such as China, fear a re-emergence of the infection through importation or as a result of early relaxation of preventive measures.^2^ It is worth differentiating between several peaks associated with the first wave of severe acute respiratory syndrome coronavirus 2 (SARS-CoV-2) pandemic and the second wave. Some scientists are attempting to extrapolate from the Spanish flu and expecting a second wave,^3,4^ whereas others who do not believe that warmer summer climates will limit the progression of the pandemic expect multiple peaks of the same wave triggered by social behavior rather than environmental factors. In addition, few believe that the virus will disappear soon, as this happened with SARS-CoV.^5^ It is clear that a cloud of uncertainty surrounds all potential future scenarios relevant to the COVID-19 pandemic. However, the threat of the surge is likely to persist until herd immunity is acquired either by natural infection or through vaccination.^6^

Whether the current pandemic will end with significant natural herd immunity in a given population depends mainly on epidemiological and immunogenic considerations. This implies that the proportion of individuals who are immune to SARS-CoV-2 crosses 1–1/R0.^6,7^ The optimal level of herd immunity for SARS-CoV-2 infection is, yet, unidentified with several estimates ranging from 20 to 90%.^6-8^ Considering that the overall level of population immunity is a dynamic figure, as it increases, the rate of transmission decreases resulting in a “flattening of the curve.”^7^ For example, as of August 30, 2020, a total of 3 611 060 cases of COVID-19 had been reported in Europe since December 31, 2019, with the death toll reaching 208 748.^9^ The first population-based seroepidemiological studies in different European countries preliminarily reported variable low levels of seropositivity, mostly ranging from 0.0% to 8.5%, as of June 30, 2020.^10^ Accordingly, it is unlikely that these levels of detectable antibodies will be sufficient for indirect protection, as the upcoming fall and winter seasons approach.^10^

Consequently, it would be faster and safer for populations to reach herd immunity by vaccination. The availability of safe and effective vaccines against COVID-19 has become one of the pillars of any program aimed at controlling the pandemic. At the same time, efforts required, challenges associated with rapidly developing, and evaluating and producing this vaccine on a large scale are enormous.

The most recent draft landscape of COVID-19 candidate vaccines, released by the World Health Organization (WHO) on August 28, 2020, listed 33 candidate vaccines in clinical evaluation and 139 candidate vaccines in preclinical evaluation from different countries, developers, research centers, and a variety of platform coronavirus targets.^11^ Regarding the stage of clinical evaluation and regulatory status of the candidate vaccines in the clinical evaluation category (n = 33), 8 products out of 33 are in Phase 3 (1 developed by the University of Oxford/AstraZeneca, 1 by Sinovac, 1 by Moderna/NIAID, 1 by Wuhan Institute of Biological Products/Sinopharm, 1 by Beijing Institute of Biological Products/Sinopharm, 1 by Gamaleya Research Institute, 1 developed by CanSino Biological Inc./Beijing Institute of Biotechnology, and 1 by BioNTech/Fosun Pharma/Pfizer), and 2 products out of 33 are in Phase 2 (1 developed by Anhui Zhifei Longcom Biopharmaceutical/Institute of Microbiology, Chinese Academy of Sciences, and the other by CureVac AG).^11^

In July 2020, results of 3 early phase COVID-19 vaccine trials in humans were reported, 1 from the messenger ribonucleic acid (mRNA)-1273 Study Group, the second from investigators at the Jenner Institute at Oxford University (Oxford, UK), with support from AstraZeneca, and the third from investigators supported by CanSino Biologics in Wuhan, China.^12-14^ All trials reported promising interim findings in human subjects where the vaccine candidates successfully induced anti–SARS-CoV-2 humoral response in more than 90% of the participants, without resulting in severe adverse events.^12-14^

Despite these efforts, a vaccine available globally by next fall is unlikely, given the time required for testing, manufacturing, and distribution that typically accompany the release of any new vaccine.^15-17^ Other mitigation methods of the pandemic will still be needed until a vaccine becomes available.

Non-pharmacological measures such as social distancing, hand hygiene, cough etiquette, quarantine and isolation of infected individuals and contacts, and travel bans have curbed the immediate increase in the number of infections in some areas.^18^ The major limitation associated with these measures is their multidimensional burden. This includes their negative effect on the health system, social life, education, political dynamics, mental well-being of the community, in general, and, perhaps most acutely, heavy burden on rich and poor economies.^1,19^ There is no doubt that dealing with the economic sequelae of the pandemic has become as important as the pandemic itself. New contingency preparedness plans should be established for the coming days that aim at applying new social norms. These plans should help mitigate new peaks of the pandemic while relaxing the social isolation rules.

These preparedness plans should include avoidance strategies for other preventable infectious diseases that might put extra burden on health systems. They should also anticipate appropriate management of comorbidities at the individual level and include other methods to support the individuals’ immune systems. These plans should be applied at the patient, public health, and hospital levels.

In this paper, we discussed recommendations that a practicing physician should provide to patients, especially those at risk of infection, as pre-emptive measures in anticipation for the potential next peak of the pandemic. In addition, a brief overview on hospital infection control measures will be highlighted.

## RECOMMENDATIONS AT THE PATIENT LEVEL

### Control of Comorbidities

Specific comorbidities have been reported among severe cases of COVID-19 in different cohort studies from countries around the world. To date, there are no studies reporting a direct causal relationship between these underlying diseases and the development of COVID-19.^20-23^ Some conditions are rather significantly associated with critical care unit admission, poor prognosis, and mortality in SARS-CoV-2-infected cases.^20-23^ The most common comorbidities associated with worsening patient outcome are hypertension, diabetes, cardiovascular disease, chronic respiratory disease, immune-compromised status, cancer, and obesity.^20-23^ The presence of more than 1 comorbidity also correlates with worse clinical outcomes among COVID-19 cases.^20-23^ Considering the rapid spread and high mortality rates associated with COVID-19, it is important and logical for 1 to control these comorbid conditions, given their strong association with severe disease and poor prognosis.

Patients with type 2 diabetes, for example, are prone to have other components of the metabolic syndrome, including hypertension and dyslipidemia, in addition to probable obesity. Therefore, continuation with an appropriate antidiabetic, antihypertensive, and lipid-lowering drug regimen in these patients is of utmost importance.^20,24-26^ Renin–angiotensin–aldosterone system blockers, including angiotensin-converting enzyme inhibitors (ACEI) and angiotensin receptor blockers (ARB), are a mainstay therapy for adequately controlling blood pressure in these patients and simultaneously preventing damage to the heart, vessels, brain, and kidneys.^25^ Regarding ACEI and ARB use, debates exist regarding the tentative risk of increased susceptibility for infection and uncertain benefit in mitigating inflammatory injury, in the absence of clear-cut evidence of a need to either continue or discontinue therapy.^20,24,25^ Nevertheless, at present, most international scientific societies and expert panels in cardiology and endocrinology have recommended the continuation of ACEI and ARB, unless explicit contraindications arise, or clear evidence emerges against their use.^20,24,25^ Regarding statins, other than their lipid-lowering activity, their cardioprotective and immunomodulatory properties should also be taken into consideration in the setting of SARS-CoV-2 infection. Thus, ACEI/ARB and statin therapy should not be discontinued.^20,24-26^

In general, for patients experiencing any type of comorbidities, clinicians should consider the following in their daily practice, as part of their COVID-19 mitigation strategies: (1) minimizing face-to-face consultations and hospital visits, especially for immune-compromised patients, through the use of virtual consultation and web-based technology to transmit important information to patients and their caregivers, in addition to dispensing medications for extended periods whenever possible; (2) prioritizing care according to a scale, taking into consideration the risk of complications with COVID-19, severity, and prognosis of underlying illness; and (3) deferring non-urgent routine evaluations and elective procedures.^20,27,28^

### The Role of Vitamins and Minerals

Some clinicians support the use of vitamin and mineral supplements to treat respiratory viral infections. Multiple ongoing studies are evaluating the use of adjunctive therapies, including vitamin and mineral supplements for both the treatment and prevention of COVID-19.

#### Vitamin C

There has always been an interest in the effect of vitamin C on respiratory infections.^29-31^ Vitamin C (ascorbic acid) is a vitamin found in fruits and vegetables, particularly citrus fruits. It is water-soluble and plays an important role in regulating body glucose metabolism. It is crucial for iron absorption, wound healing, collagen formation, and neurotransmitter production.^32^ Vitamin C is acquired through a balanced diet, but numerous dietary supplements are also available over the counter. A double-blind trial involving 818 volunteers was conducted in 1972 to assess the effect of 1 g of vitamin C taken orally every day on the frequency and duration of the common cold.^33^ In terms of the average number of colds and days of sickness per subject, the vitamin C arm experienced less illness than the placebo arm, although without statistical significance.^33^ However, regarding the number of participants who remained free of illness throughout the study period and the total days they were confined to the house or took a sick leave from work, there was a statistically significant difference between the 2 arms in favor of the vitamin C group.^33^ In a meta-analysis of 31 randomized controlled trials investigating the role of vitamin C in respiratory infection prophylaxis in 2013, Hemila and Chalker demonstrated that vitamin C supplementation reduced the incidence of the common cold in a subpopulation of patients under heavy short-term physical stress.^29^ The investigators demonstrated that vitamin C shortened the duration of the common cold in all studies.^29^ In another meta-analysis of 5 trials studying the effect of administering vitamin C in pneumonia patients in 2013, Hemila and Louhiala found that vitamin C decreased the incidence of pneumonia.^30^ A literature review published in 2017 revealed encouraging hints about the potential role of vitamin C in treating or preventing certain infections, mainly the common cold.^31^ The dose and duration of treatment, however, remains unclear, and the need for further studies was the obvious conclusion.^31^ A Cochrane review was initiated in 2019 to assess the safety and efficacy of oral vitamin C supplements taken, compared with a normal diet, in treating acute upper respiratory tract infections in adults and children.^34^

A new clinical trial (HELPCOVID-19) was recently registered to evaluate the role of hydroxychloroquine, vitamin C, vitamin D, and zinc in preventing the COVID-19 infection. Meanwhile, another clinical trial was started to evaluate the role of intravenous high-dose vitamin C in the treatment of individuals with SARS-CoV-2 infection.^35^ In conclusion, to date, there is no strong direct evidence regarding the benefit of vitamin C in the COVID-19 infection, whether in non-critically ill or critically ill settings.^36^

#### Vitamin D

The role of vitamin D in the body response to SARS-CoV-2 infection could be helpful in several ways. Vitamin D helps in the production of antimicrobial peptides in the epithelium of the respiratory tract, rendering infection and the development of symptoms less probable. It can aid in reducing the inflammatory response to infection with SARS-CoV-2.^37^

In a large systematic review and meta-analysis conducted by Martineau et al., daily or weekly supplementation with vitamin D was found to be safe and protective against acute respiratory tract infections. Those who were severely deficient experienced the most benefit, as protective effects were stronger in those with baseline 25-hydroxyvitamin D levels less than 25 nmol/L.^38^

In a cross-sectional analysis, researchers from the United Kingdom assessed the presence of a correlation between average vitamin D levels and the number of COVID-19 cases, as well as death rates, across 20 European countries.^39^ They detected significant crude relationships among levels of vitamin D, the number of COVID-19 cases, and the mortality caused by this infectious disease.^39^ Based on this circumstantial evidence, the government health agencies of the United Kingdom have recommended that people take vitamin D supplements through summer and autumn during this pandemic. Vitamin D supplementation could be especially important for older people, as they are at higher risk of poor outcome from COVID-19 and of vitamin D deficiency.^37^

In conclusion, vitamin D may play a protective role for COVID-19. In addition, treating vitamin D deficiency is also beneficial for health, in general.^36,39^ However, well-designed trials are needed to clearly elucidate its role in preventing and treating COVID-19.

#### Zinc

Zinc (Zn) is an essential element with a critical impact on the immune system.^40-44^ It regulates the proliferation, differentiation, maturation, and function of leukocytes and lymphocytes.^40-44^ Zn stimulates antiviral immunity.^41^ In the context of COVID-19, Zn is capable of inhibiting SARS-CoV RNA polymerase in vitro.^42-44^ It may suppress the activity of angiotensin-converting enzyme 2, the cellular receptor for SARS-CoV-2, and upregulate interferon α production.^42-44^ It has an anti-inflammatory effect through preventing nuclear factor-kappa B signaling and adjustment of regulatory T -cell functions.^42-44^ In addition, Zn may reduce the potential of bacterial coinfection through enhancing mucociliary clearance and possessing a direct antibacterial activity against *Streptococcus pneumoniae*.^42-44^

As a general stimulant of antiviral immunity, Zn may theoretically serve as a preventive and adjuvant therapy agent for COVID-19 by decreasing inflammation, improving mucociliary clearance, preventing ventilator-induced lung injury, and modulating antiviral and antibacterial immunity.^42-44^ The anti-inflammatory effects of Zn, although not fully studied, may play a role in the amelioration of severe COVID-19, especially in patients with multiple comorbidities.^42-44^ Given the importance of inflammation in the pathogenesis of COVID-19, whether locally in the respiratory tract in those with pneumonia or systemically as in the cytokine storm, it is logical to assume a potential adjuvant therapeutic role for Zn.^42-44^

Despite little data on Zn’s direct action on SARS-CoV-2, the antiviral activity of Zn has been described in other viral illnesses.^42-44^ In a systematic review, a significant decrease in the duration of common cold symptoms and in the incidence rate ratio of developing the common cold (0.64; 95% confidence interval: 0.47–0.88; *P* = 0.006) was observed in patients who received Zn supplementation.^45^ Daily supplementation with a dose greater than 75 mg was documented to reduce the duration of the common cold.^46^

Zn deficiency is frequently encountered in diabetics, obese individuals, the elderly, coronary artery disease patients, and those with impaired immunity.^47^ Zn is present in diets containing meat, shellfish, dairy products, bread, and cereals. The daily amount needed for adults ranges from 8 to 11 mg, depending on sex.^48^ Long-term supplementation is safe within the recommended daily allowance in healthy adults.^47^ In the context of COVID-19, doses used in registered clinical trials are variable, with a maximum daily dose of 100 mg of elemental Zn.^36^ As with vitamin D, toxicity while supplementing with oral effervescent tablets or chewable pills should be taken into consideration.

## RECOMMENDATIONS AT THE PUBLIC HEALTH LEVEL

### Childhood Vaccination

Vaccination has made a significant difference to the public well-being over the past century. However, in the global pediatric population, more than 13 million children younger than the age of 1 year did not receive any vaccines in 2018. Many of them live in countries with weak health systems.^49^ On March 26, 2020, the WHO released interim guiding principles for vaccination during the pandemic.^50^ The guidance clearly stated that all immunization services must equally consider the importance of protecting people against preventable diseases through vaccination on 1 hand and guarding the safety of the public and health workers on the other, through applying physical distancing and hygiene measures and avoiding mass gatherings.^50^ Thus, in line with COVID-19 mitigation measures, the WHO guidance recommended that countries might briefly suspend or temporarily postpone preventive immunization campaigns in areas where there is no active outbreak of a vaccine-preventable disease.^50^ Subsequently, in a news release by the WHO on May 22, 2020, at least 80 million children under the age of 1 year might be at elevated risk of diphtheria, measles, and polio in at least 68 high- and low-income countries around the world.^51^ This is a result of COVID-19 substantially disrupting or completely impeding the provision of routine life-saving immunization services on a global scale since March and April 2020.^51^ Outbreaks of these vaccine-preventable diseases, among others, are disastrous for communities and health systems already struggling against COVID-19, with regard to increasing sickness and fatality rates.^50^ Agencies including Gavi, WHO, and UNICEF are calling for a joint effort to safely deliver routine childhood vaccines and proceed with immunization campaigns against these deadly vaccine-preventable diseases.^51^ Thus, public health targets should include procurement of essential childhood vaccines and ensure that children undergo recommended catch-up vaccination as soon as possible, prioritizing those most at risk. Hospitals and vaccination clinics should be prepared for physical routing and triage to separate infants and children presenting for vaccination from those potentially infected with COVID-19.

### Influenza Vaccination

Co-circulation of COVID-19 and the influenza virus has been reported in the same community.^52-54^ Both are respiratory viruses and share similar clinical manifestations to a point that COVID-19 illness has been described as a flu-like illness.^52-54^ During the fall and winter seasons, influenza virus infection exerts pressure on health systems through increasing hospital admissions, specifically to critical care units, and accounts for substantial morbidity and mortality worldwide.^55,56^ If a double-hit of influenza and COVID-19 were to occur in the same community, there would be significant competition for hospital beds, respirators, and personal protective equipment. This would also contribute to an increased workload and psychosocial impact on health care personnel and their absenteeism.

Because both viruses could co-infect the same individual and both have similar symptoms, a high burden is also present regarding testing for COVID-19. Patients presenting with influenza symptoms would require testing for COVID-19. In this case, the strain on COVID-19 testing would be aggravated, knowing that it has thus far been a mainstay in contingency planning.

The COVID-19 pandemic has led to a global economic crisis that has affected rich and poor countries. Management of national economic resources in these circumstances has become primordial, specifically in the health care field.^57^ In an assessment model of annual public health benefits and economic importance of the influenza vaccination in 27 European countries, increasing vaccination coverage from 44% to 75% in vaccination target groups was reported to reduce additional influenza-related costs between 190 and 220 million euros per year.^58^

Based on the aforementioned arguments, the need for vaccination against influenza supersedes the need for protection of the vulnerable subsets of the community because a buildup of herd immunity against influenza viruses is needed during the COVID-19 pandemic. Herd immunity to influenza by vaccination helps decrease the burden of disease on a background status totally consumed by the pandemic. Subsequently, universal influenza vaccination is recommended, rather than prioritizing vaccination of high-risk subgroups, as recommended by the WHO.^50^

### Pneumococcal Vaccination

Superimposed bacterial pulmonary infections have been described in case reports and series of COVID-19 from several parts of the world, including China, Europe, the Philippines, and Iran.^59^ Multidrug-resistant Gram-negative bacteria were reported as a common cause of nosocomial superimposed infections. In addition, several studies reported culture-negative superinfections, probably owing to the extensive use of antibiotics for COVID-19.^59,60^

Regarding Gram-positive pathogens, *Staphylococcus aureus*, Group A Streptococcus, and *Streptococcus pneumoniae* have been reported to co-isolate with COVID-19.^59,60^ *S. pneumoniae* has been identified as the primary cause for community-acquired pneumonia and secondary bacterial infections during pandemic and seasonal influenza.^61,62^ The estimated proportion of pneumococcal co-infections among influenza deaths ranged from approximately 7% during the 2009 H1N1 pandemic to more than 50% during the 1918 pandemic.^63-65^ Invasive and noninvasive pneumococcal infections cause substantial mortality in children younger than the age of 5 years and in adults older than 65 years with or without comorbidities, leading to an extra burden on health systems, specifically critical care units.^66,67^ The hypothesis that *S. pneumoniae* can complicate COVID-19 in the winter season is plausible but has to be proven.^61^ Pneumococcal vaccination might theoretically prevent superimposed *S. pneumoniae* infection in patients with COVID-19 during the coming winter. In addition, pneumococcal vaccination mitigates the effect of pneumococcal disease and thus reduces the burden on health care regarding hospitalization rates and extra costs.^68,69^

## RECOMMENDATIONS AT THE HOSPITAL LEVEL

When preparing for a surge in the number of COVID-19 cases, increasing critical care unit bed capacity, coupled with an increase in the number of mechanical ventilation devices, is crucial.^70,71^ Budget planning for procurement of personal protective equipment and alcohol-based hand sanitizers, as well as preparedness for potential worsening staff shortage is also required for such circumstances.^71-73^ In several COVID-19 reports, nosocomial bacterial superinfections, including nosocomial pneumonia, were most commonly caused by *Pseudomonas aeruginosa*, *Acinetobacter baumannii*, and multidrug-resistant *Enterobacteriaceae*.^59^ Tight infection control measures that include applying bundles, mainly in ventilator-associated pneumonia and central line-associated bloodstream infections, have succeeded in decreasing infection rates in critical care settings.^74^ Coupling infection prevention and control measures with antibiotic stewardship additionally limits the emergence and spread of difficult-to-treat multidrug-resistant organisms.^75,76^ This is the time when rigorous implementation of infection control and antibiotic stewardship is mostly required in the preparation of a potential surge in the need for critical care beds and mechanical ventilation therapy.

## CONCLUSION

The global situation with COVID-19 during the upcoming fall is still unknown, but persistence of the virus in communities is likely. Individuals, physicians, hospitals, public health agencies, and governments should be prepared for the worst-case scenario. Control of comorbidities and supplementation with vitamin C, vitamin D, and Zn within the recommended daily allowance are logical, when extrapolated from other respiratory illnesses, while awaiting direct evidence of their effect in COVID-19. Only vitamin D supplementation has been officially recommended in the United Kingdom. We recommend catch-up childhood vaccination and universal flu vaccination. Pneumococcal vaccine in vulnerable subpopulations will also decrease the burden of this infection in hospitals, especially in critical care units. Hospitals should enhance and reinforce their infection prevention and control measures and keep a close watch on the judicious use of antimicrobials, as antimicrobial resistance could be a major contributor to the death toll due to the COVID-19 pandemic.

## List of Abbreviations

ACEI: angiotensin-converting enzyme inhibitors
ARB: angiotensin receptor blockers
COVID-19: coronavirus disease
mRNA: messenger ribonucleic acid
NIAID: National Institute of Allergy and Infectious Diseases
SARS-CoV: severe acute respiratory syndrome coronavirus
SARS-CoV-2: severe acute respiratory syndrome coronavirus 2
UNICEF: United Nations International Children’s Fund
WHO: World Health Organization
